# Licarin-B Exhibits Activity Against the *Toxoplasma gondii* RH Strain by Damaging Mitochondria and Activating Autophagy

**DOI:** 10.3389/fcell.2021.684393

**Published:** 2021-06-11

**Authors:** Jili Zhang, Hongfei Si, Kun Lv, Yanhua Qiu, Jichao Sun, Yubin Bai, Bing Li, Xuzheng Zhou, Jiyu Zhang

**Affiliations:** ^1^Ningbo University School of Medicine, Ningbo, China; ^2^Lanzhou Institute of Husbandry and Pharmaceutical Sciences, Chinese Academy of Agricultural Sciences, Lanzhou, China; ^3^Key Laboratory of New Animal Drug Project of Gansu Province, Lanzhou, China; ^4^Key Laboratory of Veterinary Pharmaceutical Development, Ministry of Agriculture, Lanzhou, China; ^5^College of Pharmacy, Jinan University, Guangzhou, China; ^6^Ningbo University School of Business, Ningbo, China

**Keywords:** natural compounds, *T. gondii*, licarin-B, proliferation, TEM

## Abstract

*Toxoplasma gondii* is an obligate intracellular pathogen that infects warm-blooded animals and humans. However, side effects limit toxoplasmosis treatment, and new drugs with high efficiency and low toxicity need to be developed. Natural products found in plants have become a useful source of drugs for toxoplasmosis. In this study, twenty natural compounds were screened for anti-*T. gondii* activity by Giemsa staining or real-time fluorescence quantitative polymerase chain reaction (qPCR) *in vitro*. Among these, licarin-B from nutmeg exhibited excellent anti-*T. gondii* activity, inhibiting *T. gondii* invasion and proliferation in a dose-dependent manner, with an EC_50_ of 14.05 ± 3.96 μg/mL. In the *in vivo*, licarin-B treatment significantly reduced the parasite burden in tissues compared to no treatment, protected the 90% infected mice from to death at 50 mg/kg.bw. Flow cytometry analysis suggested a significant reduction in *T. gondii* survival after licarin-B treatment. Ultrastructural changes in *T. gondii* were observed by transmission electron microscopy (TEM), as licarin-B induced mitochondrial swelling and formation of cytoplasmic vacuoles, an autophagosome-like double-membrane structure and extensive clefts around the *T. gondii* nucleus. Furthermore, MitoTracker Red CMXRos, MDC, and DAPI staining showed that licarin-B promoted mitochondrial damage, autophagosome formation, and nuclear disintegration, which were consistent with the TEM observations. Together, these findings indicate that licarin-B is a promising anti-*T. gondii* agent that potentially functions by damaging mitochondria and activating autophagy, leading to *T. gondii* death.

## Introduction

*Toxoplasma gondii*, which causes toxoplasmosis, is the most successful intracellular protozoan parasite that infects virtually all warm-blooded vertebrates (Weiss and Kim, 2013). Although *T. gondii* infections are generally symptomless in healthy individuals, *T. gondii* infection often results in major problems in immune-compromised individuals, such as untreated AIDS patients (Ahmadpour et al., 2014; Machala et al., 2015). Furthermore, during pregnancy, *T. gondii* infection can result in vertical transmission and lead to miscarriage, fetal malformations and even fetal death (Fallahi et al., 2018).

Currently, among the therapies of choice for toxoplasmosis, pyrimethamine, and sulfadiazine are the gold-standard chemotherapy. However, these therapeutic options are limited by poor tolerance (Rothova et al., 1993; Bosch-Driessen et al., 2002; de-la-Torre et al., 2011; Guaraldo et al., 2018). Moreover, available treatments are not effective against latent infections. Therefore, novel therapies are needed for treating toxoplasmosis. In recent years, many efforts have been focused on finding safe drugs for *T. gondii* infection. In general, screening a vast array of natural compounds is a viable option for antiparasitic drug discovery. The natural products found in plants have become a useful source of drugs for clinical use (Sepulveda-Arias et al., 2014), and some of these new compounds may serve as good starting points for the discovery of effective new drugs, such as ursolic acid, resveratrol, licochalcone A, and myrislignan (Montazeri et al., 2017; Chen et al., 2019; Guo et al., 2019; Zhang J. et al., 2019). Indeed, screening natural products might reveal viable sources of alternative therapies for parasitic infection. In this study, we screened 20 compounds comprising natural products to identify candidates that suppress the growth of *T. gondii*, and many exhibited activities against *T. gondii.* Licarin-B, a compound from spice nutmeg, showed excellent anti-*T. gondii* activity. Subsequently, we explored the activity of licarin-B against *T. gondii in vitro* and preliminarily investigated its mechanism of action.

## Materials and Methods

### Cells and Parasites

Human foreskin fibroblasts (HFFs) were obtained from the Chinese Academy of Sciences (Shanghai, China) and cultured in Dulbecco’s modified Eagle’s medium (DMEM, Gibco, United States) with streptomycin (100 μg/mL, Gibco, United States), penicillin (100 U/mL, Gibco, United States), 1% GlutaMAX (Gibco, United States), 1% non-essential amino acids (NEAAs, Gibco, United States) and 10% heat-inactivated fetal bovine serum (FBS, Gibco, United States) at 37°C in a 5% CO_2_ atmosphere (Sepulveda-Arias et al., 2014).

Tachyzoites of the *T. gondii* strain RH used in this study were a gift from the Lanzhou Veterinary Research Institute, Chinese Academy of Agricultural Sciences. The tachyzoites were maintained in HFF layers as described previously (Sepulveda-Arias et al., 2014).

### Drugs

Licochalcone B (HY-N0373), licochalcone C (HY-N0374), isoliquiritigenin (HY-N0102), licorice glycosides (HY-N6980), echinatin (HY-N0269), genistin (HY-N0595), genistein (HY-14596), glycitein (HY-N0016), D-mandelic acid (HY-Y0585), limonin (HY-17411), maslinic acid (HY-N0629), thymol (HY-N6810), chlorogenic acid (HY-N0055), myristic acid (HY-N2041), dehydrodiisoeugenol (HY-N0589), and methyl myristate (HY-Y1298) were purchased from MedChemExpress Co., Ltd. (United States), and all had purities greater than 98%. L-mandelic acid (S61263), piperitone (B29297), and myristicin (B21076) were purchased from Yuanye Biotechnology Co., Ltd. (Shanghai, China) had had purities greater than 98%. Licarin-B ([5-[(2S)-2,3-D\dihydro-7-methoxy-3β-methyl-5-[(E)-1-propenyl] benzofuran-2-yl]-1,3-benzodioxole], batch number DL0060) was purchased from Desite Biotechnology Co., Ltd. (Chengdu, China) and had a purity above 98%. Among these, some of the natural compounds of flavonoid isolates from licochalcone root, including licochalcone B, licochalcone C, isoliquiritigenin, licorice glycosides, and echinatin; genistin, genistein, and glycitein, were isolated from soybean. In addition, D-mandelic acid, L-mandelic acid, limonin, maslinic acid, thymol, piperitone, and chlorogenic acid were isolated from *Amygdalus communis* L., grapefruit, maythorn, *Piper nigrum*, and *Lonicera japonica* Thunb, and myristic acid, myristicin, licarin-B, dehydrodiisoeugenol, and methyl myristate were isolated from nutmeg. In the cytotoxicity assay, the natural compounds were dissolved in dimethyl sulfoxide (DMSO, Sigma, United States) and diluted in DMEM containing 1% FBS to different concentrations. Sulfadiazine and pyrimethamine (Sigma, United States), which were used as positive controls, were dissolved in DMEM with 1% FBS to 8.6 and 1.0 μg/mL, respectively (Neville et al., 2015).

### Ethics Statement

Ethics BALB/c mice (8 weeks, 18–20 g, female) were kept in cages with an adequate temperature (25 ± 2°C) and provided water and food. All experiments were approved by the Animal Administration and Ethics Committee of Lanzhou Institute of Husbandry and Pharmaceutical Sciences, Chinese Academy of Agricultural Sciences. The certificate number was SCXK (Gan) 2019-0012. All procedures in this study were strictly carried out accordance with good laboratory animal practice standards according to the Animal Ethics Procedures and Guidelines of the People’s Republic of China. All efforts were made to minimize animal suffering.

### Cytotoxicity Assay

The cytotoxicities of the natural compounds were determined using the previously published Cell Counting Kit-8 (CCK-8) method (Zhang J. et al., 2019). In brief, HFFs (1 × 10^4^ cells/well) were seeded in 96-well plates in a monolayer and incubated with different concentrations of the compounds in DMEM with 1% FBS; DMEM without compound was used as a control. After 24 h, CCK-8 solution (Biomake, United States) was added, and absorbance was measured at 450 nm using a Multiskan GO instrument (Thermo Fisher Scientific, MA, United States). The cell viability values are expressed as percentages of the control value (considered as 100% survival). The concentration of natural compound that had no toxicity for HFFs was used for *in vitro* screening experiments. Triplicate independent experiments were performed.

### *In vitro* Screening of Anti-*T. gondii* Activity

Human foreskin fibroblasts were seeded in 6-well plates and infected with 3 × 10^4^ fresh *T. gondii* tachyzoites. After 8 h, the natural compounds in DMEM supplemented with 1% FBS were added. Sulfadiazine and pyrimethamine were included as positive drug controls. After incubation for 24 h, the cells were washed twice with PBS, stained with Giemsa, and observed by light microscopy. In addition, total DNA was isolated from the cells using DNAiso Reagent (Takara), and a 529-bp repeat element was amplified by quantitative polymerase chain reaction (qPCR) with the following primers: Tox-F (5′-AGG AGA GAT ATC AGG ACT GTA G-3′), Tox-R (5′-GCG TCG TCT CGT CTA GAT CG-3′), and the Taqman probe Tox-TP (6-Fam CCG GCT TGG CTG CTT TTC CT BHQ1), as described in a previous study (Zhang J.L. et al., 2019). The inhibition rates against *T. gondii* proliferation for the natural compounds at concentrations with no HFF toxicity were calculated, and the inhibition rate in each treatment group was presented as the % of the control group. The experiment was repeated three times.

### Anti-Proliferation Activity of Licarin-B

Human foreskin fibroblast monolayers in 6-well plates were infected with 3 × 10^4^ parasites per well for 8 h, after which various concentrations of licarin-B (4.5, 6, 7.5, 9, 13.5, 18, 22.5, 27, 31.5, 36, 40.5, or 45 μg/mL) in DMEM supplemented with 1% FBS were added to the cells. DMEM without drugs was used as a parasite control. Sulfadiazine (0.4 μg/mL), was added under identical conditions as a positive drug control. After 24 h, the cells were washed twice with PBS, and total genomic DNA from the cells was obtained as described in a previous study (Zhang J.L. et al., 2019). The 50% effective concentration (EC_50_) of licarin-B on *T. gondii* was calculated. The results represent the means ± standard deviations (SD) from at least three independent experiments.

### Anti-Invasion Assessment of Licarin-B *in vitro*

Human foreskin fibroblasts were seeded in 6-well plates and incubated with different concentrations of licarin-B (4.5, 9, 13.5, 18, or 22.5 μg/mL), sulfadiazine (0.4 μg/mL, as a positive control) or without drugs in DMEM supplemented with 1% FBS and then infected with 3 × 10^6^ fresh parasites per well. The anti-invasion capability of licarin-B was determined by qPCR after 2 h of infection, as described in a previous study (Zhang J.L. et al., 2019). Triplicate independent experiments were performed.

### *In vivo* Anti-*T. gondii* Activity of Licarin-B

The therapeutic effect on acute infection was evaluated as described by Zhang; female BALB/c mice were divided into four groups consisting of 10 mice each, and each mouse was injected with 2 × 10^3^ tachyzoites by intraperitoneal injection. Then, the mice were treated with licarin-B (25 or 50 mg/kg⋅bw), the positive drugs (100 mg/kg⋅bw sulfadiazine, 50 mg/kg⋅bw pyrimethamine, or 15 mg/kg⋅bw folinic acid) or PBS (as control) (Si et al., 2018). Positive drugs were administered by oral administration, and licarin-B was administered by intraperitoneal injection. The treatments were administered once per day for 15 consecutive days, and the mice were observed for 15 days after treatment cessation. The survival times of the infected mice and the number of deaths were monitored and recorded twice daily for 30 days.

To evaluate the therapeutic effect of licarin-B on acute infection, mice were infected intraperitoneally with 1 × 10^4^ tachyzoites and then divided into three groups (six mice/group) for treatment with PBS (as a parasite control), licarin-B (50 mg/kg⋅bw), or a positive control drug, as described above (Zhang J.L. et al., 2019). The treatments were administered for 4 consecutive days, and then, the number of parasites in the tissue and blood samples were deduced from standard curves constructed using known numbers of tachyzoites, as previously described.

Dose safety assessment *in vivo* was performed to determine safe doses of licarin-B, as previously described. The mice were treated with the drug dosages outlined above for 30 days, and no discernible clinical toxicity was observed with any of the tested drug doses during this time period.

### Flow Cytometry Analysis

Approximately 1 × 10^6^ extracellular tachyzoites were incubated with licarin-B (18 or 36 μg/mL) in DMEM or with no drug as a control for 24 h, followed by centrifugation at 1,500 × *g* for 10 min. The samples were washed with PBS and suspended in 100 μL of binding buffer with 5 μL of annexin V-PE and 5 μL of 7-AAD dye in the dark for 20 min at 37°C. Double mixtures were acquired and analyzed by flow cytometry (BD FACSVerse, United States) (Giovati et al., 2018). The experiment was repeated three times.

### Transmission Electron Microscopy Analysis

For transmission electron microscopy (TEM) analysis, HFFs were seeded to confluence in T25 flasks and infected with 1.5 × 10^6^ *T. gondii* tachyzoites for 8 h. After the addition of 18 μg/mL licarin-B, the cells were cultured for 4, 8, or 16 h, digested with TrypLE Express for 2 min, washed twice with PBS, fixed with 2.5% glutaraldehyde at 4°C for 12 h, washed with PBS to remove glutaraldehyde and post-fixed with 1% osmium acid solution for 1.5 h in the dark. The samples were then washed with PBS, dehydrated in a graded alcohol series, dehydrated in acetone and embedded in Epon. Ultrathin sections were stained with uranyl acetate and lead citrate and observed under a transmission electron microscope (Tecnai Spirit Bio-TWIN, Thermo Fisher Scientific, FEI, United States).

### Evaluation of the Licarin-B Effect on *T. gondii* Tachyzoites in Mitochondria

Extracellular parasites (1 × 10^5^ per group) were incubated in DMEM with licarin-B (18 μg/mL) for 4, 8, or 16 h at 37°C; with licarin-B (18, 27, or 36 μg/mL) for 8 h at 37°C; or without any drug (as a control). Then, all the samples were stained with the MitoTracker Red CMXRos probe (50 nM, Invitrogen, United States) for 20 min.

(Zhang J.L. et al., 2019). The fluorescence intensity was observed by laser confocal microscopy (ZEISS LSM-800, Jena, Germany).

### Monodansylcadaverine Staining

For each sample, approximately 1 × 10^5^ tachyzoites were treated with licarin-B (18 μg/mL) in DMEM for 4, 8, or 16 h at 37°C; licarin-B (18, 27, or 36 μg/mL) in DMEM for 8 h at 37°C; or no drug (as a control) in DMEM. Then, the samples were washed with PBS (pH 7.4), suspended in monodansylcadaverine (MDC) working solution (100 μM) at 37°C for 1 h after centrifugation, and washed twice with PBS. The tachyzoites were resuspended in 500 μL of PBS, and the fluorescence in each group was visualized by laser scanning confocal microscopy.

### Evaluation of the Licarin-B Effects on *T. gondii* Tachyzoites in the Nucleus

Extracellular parasites (1 × 10^5^ per group) were incubated in DMEM with licarin-B (18, 27, or 36 μg/mL) or without any drug (as a control) for 16 h at 37°C. All the samples were stained with 4′,6-diamidino-2-phenylindole (DAPI) at 37°C for 10 min, and fluorescence intensity was observed by laser confocal microscopy.

### Statistical Analyses

The EC_50_ of licarin-B for tachyzoite growth inhibition was plotted as a function of the compound concentration *via* non-linear curve analysis using SPSS 19.0 software (SPSS Inc., Chicago, IL, United States). The results of the anti-invasion and anti-proliferation tests, the *in vivo* assays of the survival rate and parasite burden and data comparisons between the parasite control and treatment groups were statistically analyzed by one-way analysis of variance (ANOVA) using SPSS software. Differences were considered statistically significant at a *p*-value < 0.01.

## Results

### Screening of the Anti-*T. gondii* Activity of Natural Compounds *in vitro*

**FIGURE 1:**
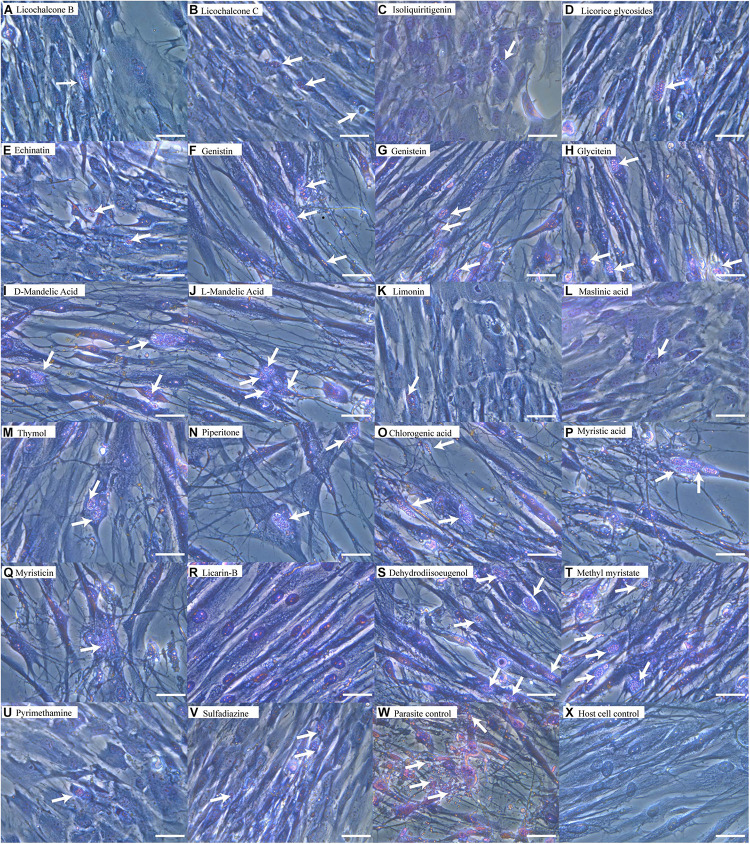
Anti-*T. gondii* activity of natural compounds as determined by Giemsa staining. HFFs were infected with *T. gondii* tachyzoites for 8 h and incubated with compounds in DMEM supplemented with 1% FBS for 24 h. The cells were washed twice with PBS, stained with Giemsa, and observed by light microscopy. Licochalcone B **(A)**, Licochalcone C **(B)**, Isoliquiritigenin **(C)**, Licorice glycosides **(D)**, Echinatin **(E)**, Genistin **(F)**, Genistein **(G)**, Glycitein **(H)**, D-mandelic acid **(I)**, L-mandelic acid **(J)**, Limonin **(K)**, Maslinic acid **(L)**, Thymol **(M)**, Piperitone **(N)** and Chlorogenic acid **(O)**, Myristic acid **(P)**, Myristicin **(Q)**, Licarin-B **(R)**, Dehydrodiisoeugenol **(S)**, Methyl myristate **(T)**, Pyrimethamine **(U)**, Sulfadiazine **(V)**, Parasite control **(W)**, Host cell control **(X)**. The arrows indicated parasitophorous vacuoles (PVs) and tachyzoites in the host cells. Scale bars: 50 μm.

The structures of the 20 natural compounds are shown in Supplementary Figure 1. To determine safe concentration ranges for these natural compounds, cytotoxicity was measured by the CCK-8 assay. Table 1 shows the ranges of the natural compound concentrations that were not toxic for HFFs, representing the safe concentration ranges for the subsequent screening of the anti-*T. gondii* activity of the compounds *in vitro* using Giemsa staining and qPCR. Accordingly, compared with the control group, licochalcone B, licochalcone C, isoliquiritigenin, licorice glycosides, echinatin, genistin, genistein, glycitein, D-mandelic acid, L-mandelic acid, limonin, maslinic acid, thymol, piperitone, chlorogenic acid, myristic acid, myristicin, licarin-B, dehydrodiisoeugenol, and methyl myristate showed activity against *T. gondii* growth, with inhibition rates of 51.67 ± 6.71, 54.91 ± 4.85, 42.82 ± 7.92, 35.93 ± 4.78, 63.06 ± 10.65, 26.33 ± 3.54, 22.32 ± 5.91, 8.11 ± 2.19, 9.30 ± 3.76, 11.87 ± 2.84, 72.19 ± 9.78, 65.44 ± 4.53, 25.66 ± 2.31, 31.14 ± 1.97, 34.56 ± 2.77, 26.33 ± 1.32, 22.33 ± 3.46, 99.76 ± 5.81, 8.14 ± 2.15, and 7.65 ± 2.64%, respectively (Table 2). Furthermore, Giemsa staining analysis revealed the corresponding dose-response anti-parasitic activities of the 20 compounds, as shown in Figure 1. Accordingly, preliminary screening results showed that licarin-B (200 μg/mL) exhibited excellent anti-proliferative activity, causing no parasitophorous vacuoles (PVs) in host cells (Figure 1R). Therefore, we further explored the anti-*T. gondii* activity of licarin-B *in vitro* and the mechanism of action.

**TABLE 1:** Ranges of natural compound concentrations that were not toxic to HFF cells.

**Compound**	**Source**	**Natural compound concentration range with no toxicity to HFF cells**
Licochalcone B	Licorice root	0.01–16 μg/mL
Licochalcone C		0.01–4 μg/mL
Isoliquiritigenin		0.01–4 μg/mL
Licorice glycosides		0.01–80 μg/mL
Echinatin		0.01–4 μg/mL
Genistin	Soybean	0.01–32 μg/mL
Genistein		0.01–40 μg/mL
Glycitein		0.01–40 μg/mL
D-mandelic acid	*Amygdalus communis* L.	0.01–80 μg/mL
L-mandelic acid		0.01–80 μg/mL
Limonin	Citrus, grapefruit, lemon, orange	0.01–80 μg/mL
Maslinic acid	Jujube, loquat leaf, maythorn, pomegranate	0.01–4 μg/mL
Thymol	*Thymus serpyllum* L., *Thymus vulgaris* L., *Origanum vulgare* L., *Trachyspermum ammi* (L.) Sprague	0.01–16 μg/mL
Piperitone	*Piper nigrum* L.	0.01–20 μg/mL
Chlorogenic acid	*Lonicera japonica* Thunb.	0.01–400 μg/mL
Myristic acid	Nutmeg	0.01–20 μg/mL
Myristicin		0.01–40 μg/mL
Licarin-B		0.01–200 μg/mL
Dehydrodiisoeugenol		0.01–80 μg/mL
Methyl myristate		0.01–160 μg/mL

*The experiment was repeated three times.*

**TABLE 2:** Screening of anti-*T. gondii* activity of natural compounds *in vitro*.

**Compound**	**Natural compound concentration for *in vitro* anti-*T. gondii* activity**	**Mean inhibition rate (% of control)**
Licochalcone B	16 μg/mL	51.67 ± 6.71
Licochalcone C	4 μg/mL	54.91 ± 4.85
Isoliquiritigenin	4 μg/mL	42.82 ± 7.92
Licorice glycosides	80 μg/mL	35.93 ± 4.78
Echinatin	4 μg/mL	63.06 ± 10.65
Genistin	32 μg/mL	26.33 ± 3.54
Genistein	40 μg/mL	22.32 ± 5.91
Glycitein	40 μg/mL	8.11 ± 2.19
D-mandelic acid	80 μg/mL	9.30 ± 3.76
L-mandelic acid	80 μg/mL	11.87 ± 2.84
Limonin	80 μg/mL	72.19 ± 9.78
Maslinic acid	4 μg/mL	65.44 ± 4.53
Thymol	16 μg/mL	25.66 ± 2.31
Piperitone	20 μg/mL	31.14 ± 1.97
Chlorogenic acid	400 μg/mL	34.56 ± 2.77
Myristic acid	20 μg/mL	26.33 ± 1.32
Myristicin	40 μg/mL	22.33 ± 3.46
Licarin-B	200 μg/mL	99.76 ± 5.81
Dehydrodiisoeugenol	80 μg/mL	8.14 ± 2.15
Methyl myristate	160 μg/mL	7.65 ± 2.64

*The inhibition rates of the natural compounds on *T. gondii* proliferation were determined by qPCR, and the inhibition rate in each treatment group is presented as the % of the control group. The experiment was repeated three times.*

### Licarin-B Inhibited *T. gondii* Tachyzoite Proliferation and Invasion

**FIGURE 2:**
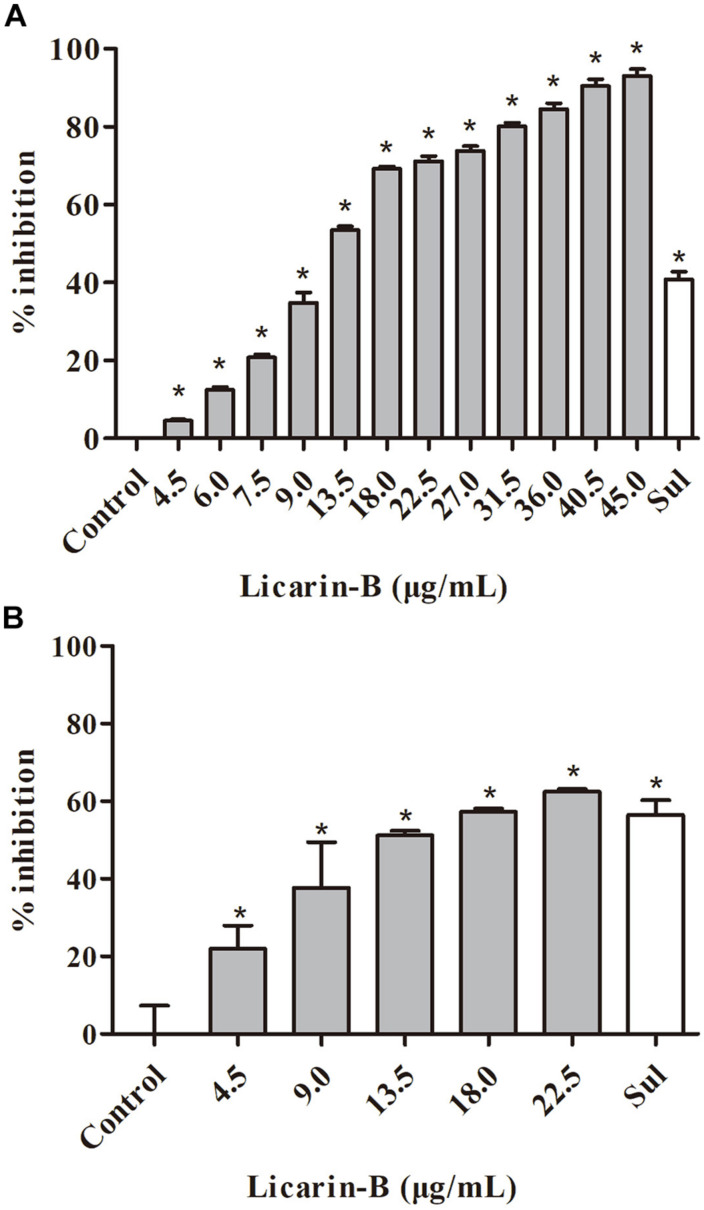
Licarin-B inhibited *T. gondii* tachyzoite intracellular proliferation and invasion *in vitro*. **(A)** Assessment of the anti-proliferative effect of licarin-B on *T. gondii*-infected HFFs by qPCR. HFFs were infected with *T. gondii* and then cultured in DMEM with licarin-B (4.5, 6, 7.5, 9, 13.5, 18, 22.5, 27, 31.5, 36, 40.5, or 45 μg/mL), sulfadiazine (0.4 μg/mL), or no drug (parasite control). After 24 h, a 529-bp repeat element of the *T. gondii* genome was amplified by PCR. The percent inhibition of tachyzoite proliferation in each group was compared with that in the parasite control group. **(B)** Effects of licarin-B on *T. gondii* invasion. HFFs were cultured in DMEM containing licarin-B (4.5, 9, 13.5, 18, or 22.5 μg/mL), sulfadiazine (0.4 μg/mL), or no drug (parasite control) and infected with *T. gondii* tachyzoites. The anti-invasion capability of licarin-B was determined by qPCR after 2 h of infection. The percentage of invasive cells is expressed as the number of infected cells compared with the total number of host cells. Triplicate independent experiments were performed, and the data are presented as the mean ± SD. *p < 0.01 compared with the parasite control.

The ability of licarin-B to suppress the intracellular proliferation of *T. gondii* tachyzoites was evaluated by qPCR. Compared with the control group, licarin-B significantly (*p* < 0.01) inhibited *T. gondii* tachyzoite intracellular proliferation in a concentration-dependent manner in the range of 4.5–45 μg/mL (Figure 2A). The EC_50_ of licarin-B for *T. gondii* tachyzoite growth inhibition was 14.05 ± 3.96 μg/mL. Sulfadiazine (0.4 μg/mL), as the positive control drug, inhibited *T. gondii* tachyzoite intracellular proliferation by 40.82%.

In the anti-invasion assay, compared with the control group, licarin-B (4.5–22.5 μg/mL) significantly (*p* < 0.01) inhibited *T. gondii* invasion (Figure 2B). The anti-invasion inhibition rate of the positive drug (sulfadiazine) control group was 56.5%.

### Licarin-B Exhibited Anti-*T. gondii* Activity *in vivo*

**FIGURE 3:**
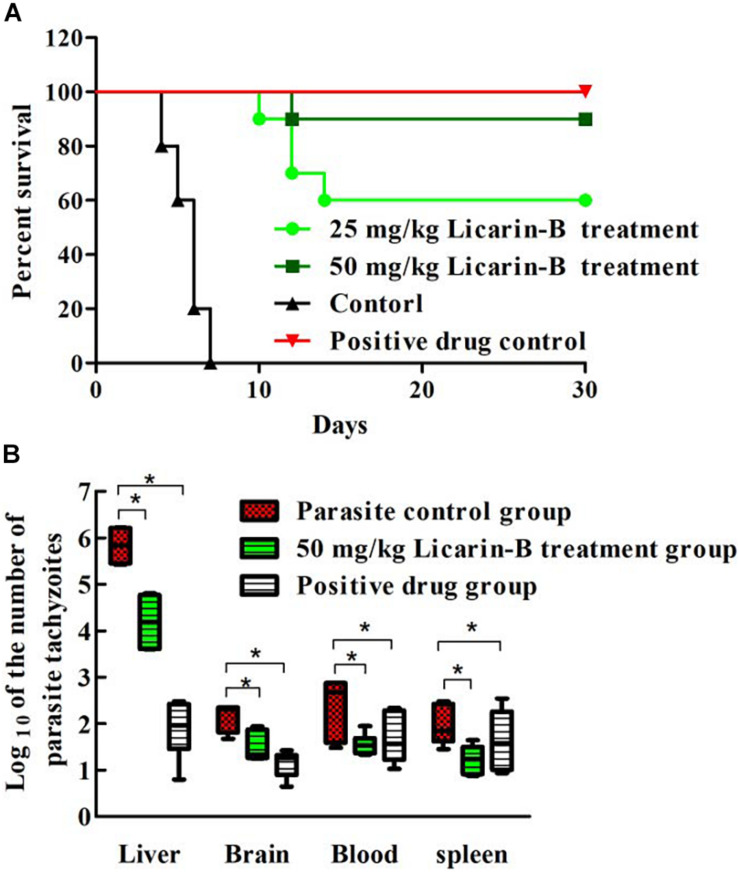
Effect of licarin-B on the survival rate of acutely infected mice **(A)**. All the mice were infected with *T. gondii* tachyzoites and then treated with licarin-B (25 or 50 mg/kg⋅bw), a positive control drug PBS once daily for 15 days. The mice were then observed for an additional 15 days, and the survival times of the infected mice were recorded for 30 days. Licarin-B treatment reduced the parasite burden in blood or tissues from the acutely infected mice **(B)**. The mice were challenged intraperitoneally with *T. gondii* tachyzoites and then treated with licarin-B or PBS once daily for 7 days. The parasite loads in the blood and brain, liver, and spleen tissues of the infected BALB/c mice were isolated and homogenized. Total genomic DNA was isolated, and the *T. gondii* 529-bp gene was detected by qPCR. The quantified parasite loads in the tissues and blood of mice are presented as the log10 values of the numbers of tachyzoites per 20 mg of tissue or 50 μL of blood. *p < 0.01 compared with the parasite control.

To determine the effect of licarin-B on the survival rate of acutely infected mice, *T. gondii*-infected mice were treated with licarin-B during a 30-day test period. All the infected mice in the parasite control and solvent-treated groups succumbed to infection by day 7, as shown in Figure 3A. Licarin-B had a significant (*p* < 0.01) anti-*T. gondii* activity, as demonstrated by survival rates of 60 and 90% of the infected mice after the 15-day period of continuous administration 25 and 50 mg/kg⋅bw, respectively. All the mice in the positive control drug groups survived. Licarin-B and the positive control drug treatments significantly (*p* < 0.01) reduced the parasite loads in the blood and brain, liver, and spleen tissues compared with those found in the parasite control untreated group, as shown in Figure 3B.

### Licarin-B Induced *T. gondii* Death

**FIGURE 4:**
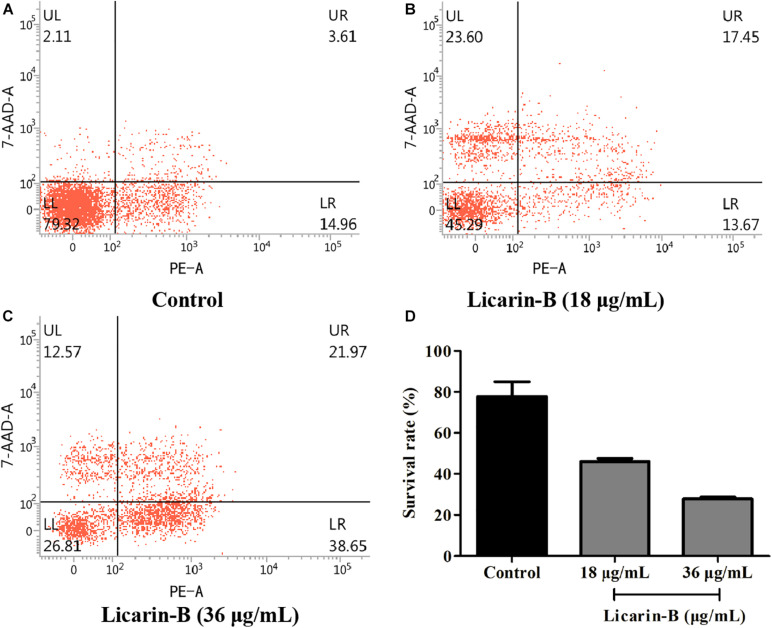
Licarin-B induced death in *T. gondii*, as determined by flow cytometry. The control group showed a *T. gondii* survival rate of approximately 80% **(A)**. Licarin-B treatment significantly reduced the *T. gondii* survival rate, with rates of approximately 45 and 26% in the 18 and 36 μg/mL licarin-B treatment groups, respectively **(B,C)**. The proportions of *T. gondii* surviving after treatment with licarin-B (18 or 36 μg/mL) for 24 h are shown **(D)**.

To determine whether treatment with licarin-B was correlated with *T. gondii* death, the parasite survival rate was monitored by flow cytometry analysis. As shown in Figure 4, licarin-B treatment significantly (*p* < 0.01) reduced *T. gondii* survival, as indicated by rates of approximately 45 and 26% in the 18 and 36 μg/mL licarin-B treatment groups, respectively (Figures 4B,C). Conversely, the control group showed a survival rate of approximately 80% (Figure 4A). The proportions of *T. gondii* surviving after treatment with licarin-B (18 or 36 μg/mL) or not for 24 h are shown in Figure 4D.

### Ultrastructural Changes in *T. gondii* Tachyzoites After Licarin-B Treatment

**FIGURE 5:**
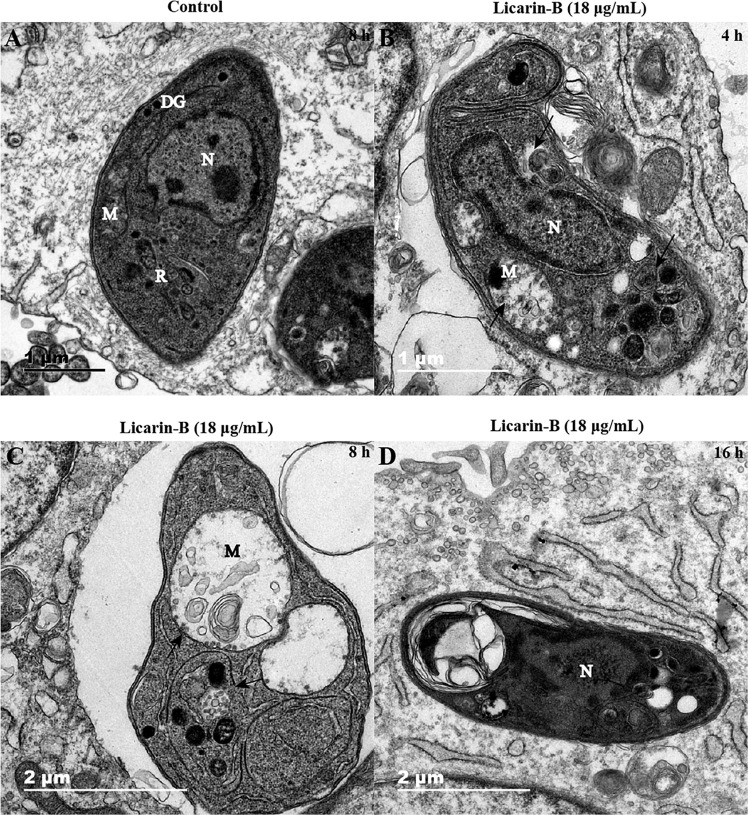
Ultrastructural changes in *T. gondii* tachyzoites after licarin-B. HFFs were infected with 1.5 × 10^6^ *T. gondii* tachyzoites; 18 μg/mL licarin-B was then added, and the cells were cultured for 4, 8, or 16 h. In the control group, the well-preserved tachyzoite structures were maintained, including the rhoptries (R), dense granules (DGs), nucleus (N), and mitochondrion (M) **(A)**. Licarin-B treatment of the parasites for 4 h induced cytoplasmic alterations, such as slight mitochondrial swelling and a double membrane structure **(B)**. After 8 h of incubation, licarin-B induced aggravated mitochondrial swelling, disrupted cristae, vacuole formation, and autophagy-like double membrane structures in the cytoplasm **(C)**. Furthermore, extensive clefts around the nucleus, nuclear membrane disappearance, and progressive degeneration of the parasites were observed after 16 h incubation with licarin-B **(D)**. Scale bars: 1 μm **(A,B)**; 2 μm **(C,D)**.

Transmission electron microscopy analysis showed that the control groups displayed well-preserved tachyzoite structures, including rhoptries (R), dense granules (DGs), nuclei (N), and mitochondria (M) with an electron-dense matrix (Figure 5A). In contrast, after incubation with licarin-B (18 μg/mL) for 4 h, the parasites exhibited alterations in the tachyzoite cytoplasm, such as slight mitochondrial swelling and a double membrane structure (a hallmark of autophagy), as shown by the arrow in Figure 5B. After 8 h of incubation, licarin-B (18 μg/mL) induced mitochondrial swelling, cristae disruption, vacuole formation, and an autophagy-like double membrane structure in the cytoplasm, as indicated by the arrows (Figure 5C). Furthermore, extensive clefts around the nucleus, nuclear membrane disappearance, and progressive degeneration of the parasites were observed after 16 h of incubation with licarin-B (18 μg/mL), as depicted in Figure 5D.

### Licarin-B Induced Mitochondrial Damage in *T. gondii*

**FIGURE 6:**
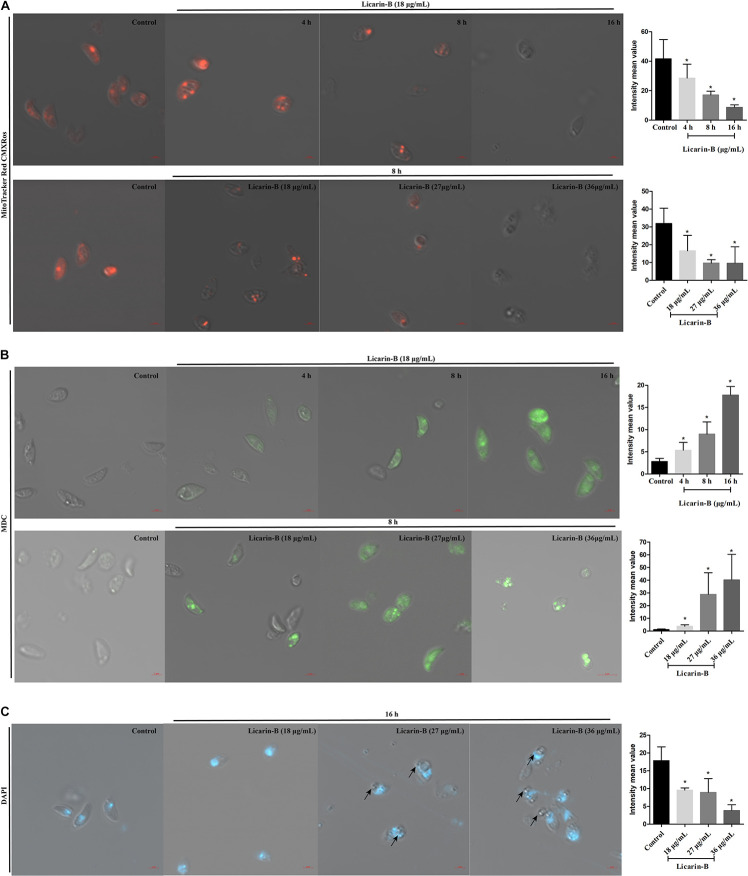
Licarin-B induced mitochondrial damage in *T. gondii* **(A)**. *T. gondii* were incubated with licarin-B (18 μg/mL) for 4, 8, or 16 h and with licarin-B (18, 27, or 36 μg/mL) for 8 h or without any drug (as a control). Then, all the samples were stained with the MitoTracker Red CMXRos and observed by laser confocal microscopy. Licarin-B induced autophagy formation in *T. gondii* **(B)**. Tachyzoites were treated with licarin-B (18 μg/mL) for 4, 8, or 16 h; with licarin-B (18, 27, or 36 μg/mL) for 8 h at 37°C; or with no drug (as a control). They were then stained with MDC working solution (100 μM), and visualized by laser scanning confocal microscopy. Licarin-B induced nuclear disintegration **(C)**. *T. gondii* were treated with licarin-B (18, 27, or 36 μg/mL) or without drug as a control for 16 h and stained with DAPI; the fluorescence intensity was observed by laser confocal microscopy. Scale bars: 2 μm. The intensity mean value of MitoTracker Red CMXRos, MDC, or DAPI staining were calculated. *p < 0.01 compared with the parasite control.

The MitoTracker Red CMXRos dye shows red fluorescence at high mitochondrial membrane potential (ΔΨm), and the change in its fluorescence color is thus considered an indicator of the mitochondrial state. *T. gondii* tachyzoites had complete ΔΨm in the control group without licarin-B incubation, and licarin-B decreased the ΔΨm in *T. gondii* in a time-dependent manner in the presence of licarin-B (18 μg/mL) for an extended period (Figure 6A). As the licarin-B concentration increased (18, 27, or 36 μg/mL), the ΔΨm was reduced in a dose-dependent manner after treatment for 8 h, as shown in Figure 6A.

### Licarin-B Induced Autophagy Formation in *T. gondii*

To verify autophagy formation after licarin-B incubation, we performed labeling experiments with MDC. After 4, 8, or 16 h of treatment with licarin-B (18 μg/mL), some parasites were positive for MDC, which labels autophagosomes (Figure 6B). Furthermore, the labeled tachyzoites showed intense green fluorescence in the groups treated with 18, 27, and 36 μg/mL licarin-B for 8 h, which indicated that autophagy was induced in *T. gondii* in a concentration-dependent manner (Figure 6B). However, MDC staining was negative in the control group (Figure 6B). Together, these results were consistent with the TEM analysis.

### Licarin-B Induced Nuclear Disintegration in *T. gondii*

To verify nuclear damage after licarin-B incubation, DAPI staining revealed a diffuse fluorescent signal in the parasite nuclei after licarin-B (18, 27, or 36 μg/mL) treatment for 16 h (Figure 6C), as indicated by the arrows, depicting a disaggregated nucleus. *T. gondii* exhibited morphological changes, such as membrane blebs, as indicated by the arrows, which are usually seen in *T. gondii* apoptosis or autophagy. The control group displayed a normally dense fluorescent signal (Figure 6C).

## Discussion

Natural products have proven useful as core sources of novel compounds, and the discovery and development of anti-*T. gondii* drugs support the function of strong anti-*T. gondii* activity of natural products (Guo et al., 2019). In this study, the screening of a vast array of natural compounds was used as an approach to identify new therapeutic targets for the treatment of *T. gondii* infection.

The safe concentration ranges of these natural compounds were evaluated by a cytotoxicity assay, and the maximum concentrations of the natural compounds that were safe for HFFs were used in anti-*T. gondii* assays *in vitro* with Giemsa staining or qPCR. Most natural compounds inhibited *T. gondii* intracellular proliferation to varying degrees. However, after incubation of the natural compounds at the maximum safe dose, *T. gondii* survival and PV formation still occurred in all treatment groups except for the licarin-B group. Licarin-B is a natural compound from nutmeg, the seeds of *Myristica fragrans* Houtt (Myristicaceae), known as the Spice Islands (Van Grol et al., 2010). Licarin-B has excellent anti-*T. gondii* activity, clearing almost all parasites from host cells. Recent studies report that some compounds from nutmeg have anti-*T. gondii* activity; for example, nutmeg essential oil and myrislignan exert anti-*T. gondii* activity, with EC_50_ values of 24.45 and 32.41 μg/mL, respectively (Pillai et al., 2012; Zhang J. et al., 2019). In comparison, licarin-B showed an EC_50_ of 14.05 ± 3.96 μg/mL for intracellular replication, indicating that the anti-*T. gondii* activity of licarin-B was better than that of myrislignan. Furthermore, 50 mg/kg.bw licarin-B exhibited significant anti-*T. gondii* activity, resulting in a 90% survival rate in mice upon acute infection, which is higher than that for myrislignan, which resulted in a 50% survival rate at 50 mg/kg.bw. The parasite burdens in the liver, spleen, brain and blood after licarin-B treatment were significantly decreased compared with those in the parasite control group. The cytotoxicity of licarin-B was similar to that of myrislignan in our previous study. Taken together, the findings indicate that licarin-B inhibits *T. gondii* tachyzoite proliferation and reduces tachyzoite cell invasion. Subsequently, flow cytometry analysis showed that licarin-B also caused *T. gondii* death in a dose-dependent manner. However, the mechanism of action of licarin-B against *T. gondii* was not clear.

Recently, natural compounds, such as ursolic acid, resveratrol, and licochalcone A, have provided the most important sources of lead compounds in the discovery of anti-*T. gondii* drugs. Resveratrol directly inhibits *Toxoplasma* activity by reducing the population of extracellularly grown tachyzoites, probably by disturbing the redox homeostasis of the parasites (Chen et al., 2019). Furthermore, (+)-usnic acid derivatives exert a strong antioxidant effect against *T. gondii* infection (Guo et al., 2019), and licochalcone A may exert an anti-*T. gondii* effect by interfering with its lipid metabolism (Si et al., 2018). Myrislignan has potent anti-*T. gondii* activities both *in vitro* and *in vivo*, and these activities might involve the interruption of mitochondrial function (Zhang J. et al., 2019). To explore the possible mechanism of action of licarin-B on *T. gondii*, TEM analysis was used. Herein, licarin-B induced mitochondrial swelling, an autophagy-like double membrane structure, cytoplasmic vacuoles, and nuclear disintegration. Subsequently, we confirmed that licarin-B reduced the ΔΨm in a time- and dose-dependent manner using the MitoTracker Red CMXRos probe. Notably, a critical difference between *T. gondii* and mammalian cells is that *T. gondii* has a single, double-membraned conventional protozoan mitochondrion, which is the powerhouse of *T. gondii*, and respiration and oxidative phosphorylation occur in *T. gondii* mitochondria (Bhatia-Kissova and Camougrand, 2010; Narendra and Youle, 2011; Weiss and Kim, 2013). Therefore, mitochondria are required for the survival of *T. gondii* (Melo et al., 2000; Ghosh et al., 2012). Studies have indicated that mitochondrial damage can lead to autophagic death in *T. gondii* (Ghosh et al., 2012). Furthermore, MDC staining and DAPI staining confirmed autophagy formation and nuclear disintegration, which usually accompanies apoptosis or autophagy in *T. gondii*. These results were consistent with the TEM analysis. Recently, a report showed that monensin kills *T. gondii* by inducing autophagy, a novel mechanism of cell death in response to an antimicrobial drug (Lavine and Arrizabalaga, 2012). In the present, we also investigated licarin-B-induced death in *T. gondii*. Given the results, licarin-B may induce autophagy by damaging the mitochondria, eventually leading to *T. gondii* death and nuclear disintegration.

## Conclusion

In conclusion, all 20 natural compounds tested exhibited a certain degree of anti-*Toxoplasma* activity, but licarin-B had a strong anti-*Toxoplasma* effect. Through an in-depth study, it was found that licarin-B can inhibit the invasion and proliferation of *T. gondii in vitro*, and also protect the infected mice from to death, reduce the parasite load in tissues and blood. Licarin-B may cause the death of *T. gondii* by damaging mitochondria and activating autophagy. Further studies are necessary to explore the mechanism of action.

## Data Availability Statement

The original contributions presented in the study are included in the article/Supplementary Material, further inquiries can be directed to the corresponding author/s.

## Ethics Statement

The animal study was reviewed and approved by JYZ and JLZ was conducted between April to May 2019 in Lanzhou Institute of Husbandry and Pharmaceutical Sciences of Lanzhou, Gansu Province, China. All experimental methods, animal care, and the barn environment of this study strictly complied with the Guide for the Care and Use of Laboratory Animals, Lanzhou Institute of Husbandry and Pharmaceutical Sciences, China. In addition, all efforts were made to minimize suffering. Thus, we agreed to conduct the experiment, and the certificate number was SCXK (Gan) 2019–0012.

## Author Contributions

HFS, XZZ, YHQ, YBB, KL, and BL revised the manuscript. JYZ directed the project and reviewed the manuscript. JLZ supervised the experiments and wrote the manuscript. All the authors read and approved the final manuscript.

## Conflict of Interest

The authors declare that the research was conducted in the absence of any commercial or financial relationships that could be construed as a potential conflict of interest.

## Supplementary Material

Supplementary Figure 1 Structures of the 20 natural compounds.
