# Supplementary figures and images for: Licarin-B Exhibits Activity Against the Toxoplasma gondii RH Strain by Damaging Mitochondria and Activating Autophagy
Source: Front Cell Dev Biol. 2021 Jun 11;9:684393. doi: 10.3389/fcell.2021.684393 (PMC8226262; doi:10.3389/fcell.2021.684393)

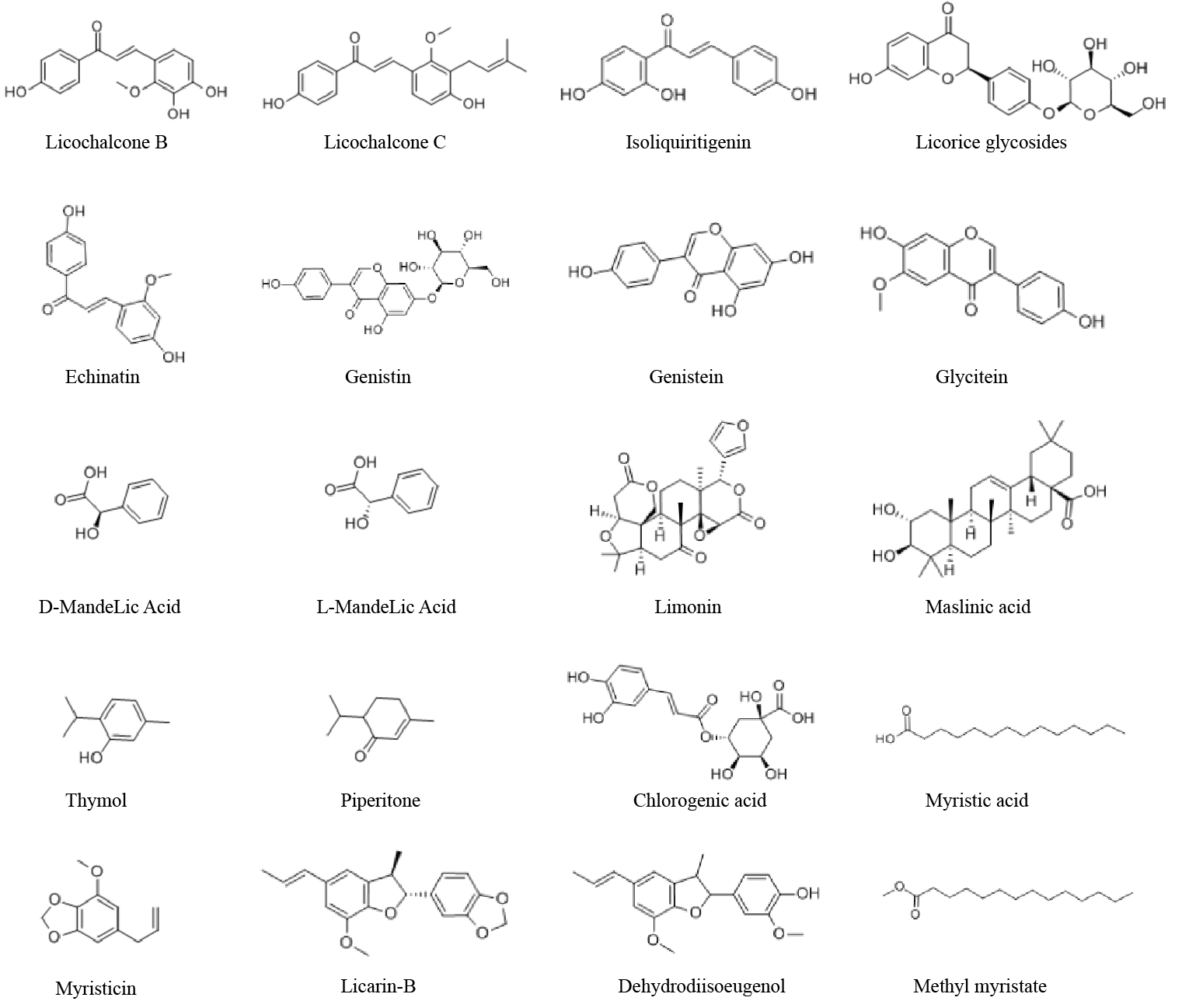

Supplement: Supplementary Figure 1 — Structures of the 20 natural compounds. [file Image_1.TIF]
